# Socioeconomic disparities in abdominal obesity over the life course in China

**DOI:** 10.1186/s12939-018-0809-x

**Published:** 2018-07-05

**Authors:** Panpan Zhao, Xiaoli Gu, Dongfu Qian, Fan Yang, for the Healthy Aging and Development Study Group in Nanjing Medical University, and for the Data Mining Group of Biomedical Big Data in Nanjing Medical University

**Affiliations:** School of Health Policy & Management, Nanjing Medical University, 101 Longmian Avenue, Jianging Districe, Nanjing, 211166 Jiangsu Province People’s Republic of China

**Keywords:** Abdominal obesity, China, Life course, Socioeconomic disparities

## Abstract

**Background:**

Abdominal obesity has become an important public health issue in China. Socioeconomic disparities are thought to be closely related to the prevalence of abdominal obesity. Exploring socioeconomic disparities in abdominal obesity over the life course in China could inform the design of new interventions to prevent and control abdominal obesity.

**Methods:**

The China Health and Nutrition Survey (CHNS) was a prospective household-based study involving seven rounds of surveys between 1993 and 2011. Twenty three thousand, two hundred and forty-three individuals were followed up over an 18-year period. The mixed effects models with random intercepts were used to assess the effects on abdominal obesity. Six key socioeconomic indicators, with age and age-squared added to the models, were used to identify socioeconomic disparities in abdominal obesity over the adult life course.

**Results:**

Prevalence of abdominal obesity increased non-linearly with age over the adult life course. Abdominal obesity was more prevalent in younger than older birth cohorts. Positive period effects on the prevalence of abdominal obesity were substantial from 1993 to 2011, and were stronger among males than females. Prevalence of abdominal obesity was higher among ethnic Han Chinese and among the married [coefficient (95% confidence intervals): 0.03(0.003, 0.057) and 0.035(0.022, 0.047), respectively], and was lower among males [coefficient (95% confidence intervals): − 0.065(− 0.075,-0.055)]. A higher-level of urbanization and higher household income increased the probability of abdominal obesity [coefficient (95% confidence intervals): 0.160(0.130, 0.191), 3.47E^− 4^ (2.23E^− 4^, 4.70E^− 4^), respectively], while individuals with more education were less likely to experience abdominal obesity [coefficient (95% confidence intervals): − 0.222 (− 0.289, − 0.155)] across adulthood.

**Conclusions:**

In China, abdominal obesity increased substantially in more recent cohorts. And people with lower educational attainment, with higher household income, or living in more urbanized communities may be the disadvantaged population of abdominal obesity over the adult life course. Effective interventions targeting the vulnerable population need to be developed.

**Electronic supplementary material:**

The online version of this article (10.1186/s12939-018-0809-x) contains supplementary material, which is available to authorized users.

## Background

Abdominal obesity has become an important public health issue in China [1]. With rapid changes in social and economic conditions, as well as dietary patterns, China has been experiencing a substantial increase in the prevalence of abdominal obesity [1]. Abdominal obesity increases risks of diabetes, metabolic syndrome [2], cardiovascular disease and mortality [3, 4]. Socioeconomic disparities are thought to be closely related to the distribution of abdominal obesity [5, 6], abdominal obesity may tend to be more prevalent among the adult population with low socioeconomic status. Exploring socioeconomic disparities in abdominal obesity could inform the design of new interventions to prevent and control abdominal obesity.

Although some studies based on cross-sectional surveys have examined socioeconomic disparities in abdominal obesity [7, 8], they could not observe the long-term changes of the variables because some socioeconomic determinants are likely to change with age and period [9, 10]. Those cross-sectional studies also lack the ability to examine cohort effects on abdominal obesity and disentangle the effects of lifespan [11] in socioeconomic disparities of abdominal obesity. So, longitudinal studies with multiple birth cohorts are needed to represent a longitudinal trend of abdominal obesity and more precisely identify such socioeconomic disparities in abdominal obesity over the adult life course. To date, longitudinal research on abdominal obesity and its socioeconomic factors with a large representative sample does not exist in China. Furthermore, waist-to-height ratio (WHtR) is regarded as a superior indicator of abdominal obesity [12], and has been shown to be a better predictor of metabolic syndrome [2], adverse cardiovascular events and mortality [3] than waist circumference (WC) or body mass index (BMI) in the general population [13, 14]. In studies that use WHtR as an indicator, abdominal obesity is generally defined as WHtR of over 0.5 [12, 13].

Data from the China Health and Nutrition Survey (CHNS) with a large population-based cohort over an 18-year period were used to assess age-period-cohort effects on abdominal obesity, based on WHtR cutoffs, to identify socioeconomic disparities in abdominal obesity over the adult life course.

## Methods

### Data sources

We used longitudinal data from the CHNS of1993, 1997, 2000, 2004, 2006, 2009, and 2011 [10, 15–17] for our analysis. The survey protocols and process for obtaining informed consent were approved by the institutional review committees of the University of North Carolina at Chapel Hill, National Institute of Nutrition and Food Safety, Chinese Center for Disease Control and Prevention, China-Japan Friendship Hospital, and Ministry of Health. The CHNS covered approximately 56% of China’s population across nine diverse provinces (Heilongjiang, Liaoning, Guangxi, Guizhou, Hubei, Hunan, Henan, Jiangsu, and Shandong). A multistage, random cluster sampling process [10] was used to draw a sample in each of the provinces. Counties and cities in each province were stratified by income (low, middle and high) and a weighted sampling scheme was used to randomly select four counties and two cities in each province. Villages and townships within the counties, and urban and suburban neighborhoods within the cities were selected randomly. In each community, 20 households were randomly selected and all household members were interviewed. The CHNS followed up the originally sampled households and new households formed from previous households by the household roster. Additional details about sampling methods, response rates, and data quality are reported elsewhere [17].

Data was collected at the local clinic or participants’ homes by well-trained health workers. Height was measured to the nearest 0.1 cm using SECA 206 wall-mounted metal tapes according to a standard protocol [18]. Waist circumference (WC) was measured to the nearest 0.1 cm using a Seca tape measure (Seca North America, Chino, CA, USA) at the midpoint between the lowest rib margin and the iliac crest. Participants were asked to remove bulky clothing and shoes. WHtR is defined as their WC (cm) divided by height (cm). Abdominal obesity was defined as the WHtR of over 0.5 [12, 13].Community urbanicity was calculated by an urbanization index that comprises a 12-multicomponent (population density, economic activity, housing, education, diversity, modern markets, traditional markets, communications, transportation infrastructure, social services, sanitation, and health infrastructure) continuous scale ranging from 0 to 120 with higher values indicating higher levels of urbanicity for the sampled communities in each survey year [19, 20].

Since WC was not measured in the 1989 and 1991 surveys, our study used data from the seven CHNS conducted between 1993 and 2011, if height (in cm) and WC (in cm) information was collected. The final analytic data contained 23,243 individuals (65,359 observations) aged of 18 and or above at multiple exams (mean number of measurements: 3). We added gender (a dummy variable coded 100 for males and 0 for females), race/ethnicity (100 for Han ethnicity and 0 for other minorities), marital status (100 for Married and 0 for others), community urbanicity, years of education and per capita net annual household income, as well as age and age-squared, into the mixed-effects models to identify socioeconomic disparities in prevalence of abdominal obesity over the adult life course [20].We chose to use mixed effects models because they are particularly useful in analyzing the relationship between the independent variables and the response variables for longitudinal settings with repeated measurements of the same statistical units [10, 20]. In three mixed effects models (Additional file 1; model 1, model 2 and model 3), we used a sample of 23,243 individuals (65,359 observations) aged 18 or above for analysis. Besides that, there were 1493 participants with measurements for all seven surveys. We used this sample of 1493 individuals to estimate the trajectories of probability of abdominal obesity prevalence across the adult life course by unadjusted mixed effects models stratified by baseline age group (birth cohort) [10]. Birth cohorts were stratified into 5 groups: Cohort 1931–1940, Cohort 1941–1950, Cohort 1951–1960, Cohort 1961–1970, and Cohort 1971–1980 (Additional file 1: Table S4).All statistical tests were conducted, using STATA version 12.

## Results

**Fig. 1:**
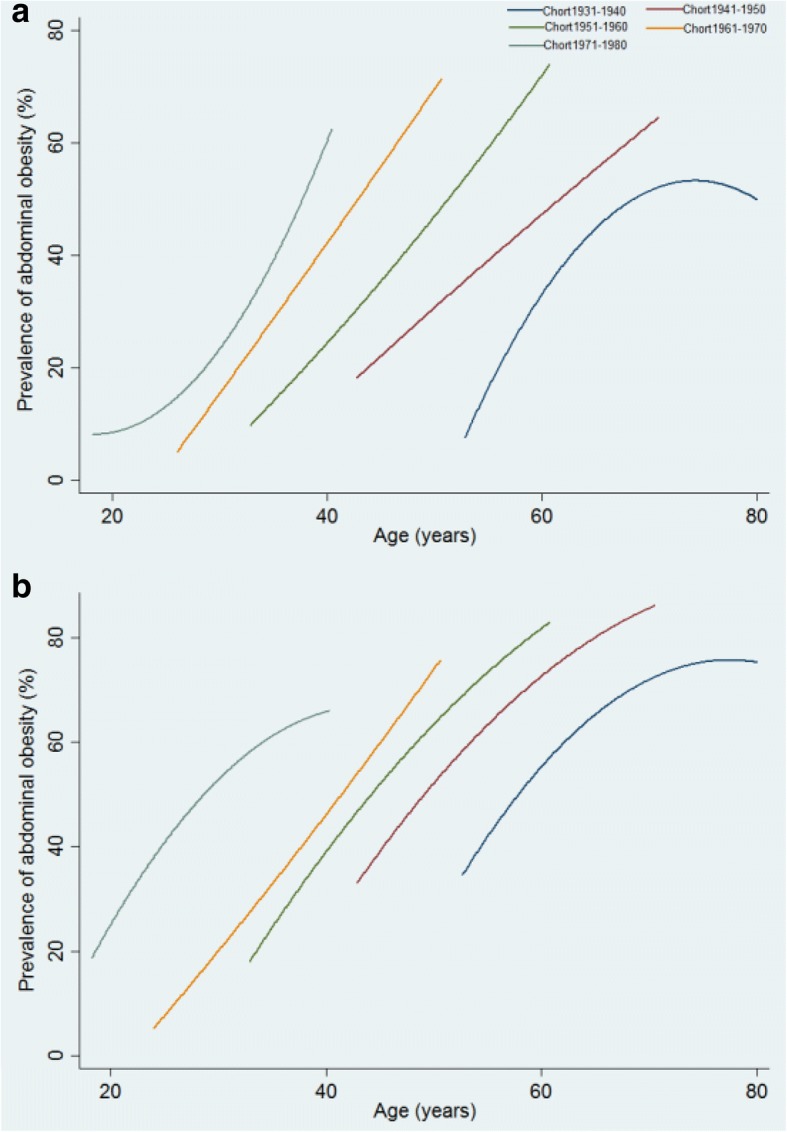
Trajectories of the probability of abdominal obesity (%) across the life course for 1493 participants with measurements for all 7 surveys among men (**a**) and women (**b**) adult, estimated by multilevel mixed effects models stratified by baseline age group (Birth cohort) (Birth cohorts were stratified into 5 groups: Cohort 1931–1940, Cohort 1941–1950, Cohort 1951–1960, Cohort 1961–1970, Cohort 1971–1980)

Table 1 showed the basic information of the participants in the 1993–2011 CHNS. Curvilinear age effects on the prevalence of abdominal obesity were observed confirming non-linear increase with age [coefficient (95% confidence intervals): 2.60(2.46, 2.74) for age and − 0.018 (− 0.019, − 0.017) for age-squared; all *P* < 0.001] over the adult life course (Table 2, Model 1, Additional file 1: Table S1). After controlling for age, significant period effects on the prevalence of abdominal obesity were observed which were particularly substantial from 1993 to 2011 (Table 2, Model 1). Unadjusted linear mixed effects model stratified by birth cohort showed that prevalence of abdominal obesity was higher in the younger birth cohorts than in the older ones (Fig. 1, Additional file 1: Table S4), and prevalence of abdominal obesity was higher in the 1941–1950, 1951–1960, 1961–1970 and 1971–1980 cohorts than that in the 1931–1940 cohort [coefficient (95% confidence intervals): 8.328(4.011, 12.644), 15.500(10.851, 20.149), 20.740(15.254, 26.225) and 34.908(24.079, 45.737), respectively; all *P* < 0.001]. And at any given age, prevalence of abdominal obesity increased for each successive cohort for both men and women (Fig. 1). For instance, at the age of 60, prevalence of abdominal obesity among men was higher in the 1941–1950 cohort than in the 1931–1940 cohort (about 30% vs 40%), but lower than in the 1951–1960 cohort (nearly 60%).

**Table 1:** General characteristic of Chinese adult population from the 1993–2011 CHNS^a^

	Survey Year						
	1993	1997	2000	2004	2006	2009	2011
Participated	7810	4498	5918	6408	6623	6319	7319
New participated	–	3865	3421	2574	2239	3067	5298
With drowal /Died	–	3312	2445	2931	2359	2543	2067
Age	42.1(15.7)	43.7(15.8)	45.2(15.4)	48.2(15.3)	49.5(15.2)	50.4(15.4)	51.3(15.2)
Gender^b^	47.4	48.5	47.7	47.5	46.9	47.3	46.7
Ethnicy^c^	95.4	94.5	95.1	95.2	95.7	95.7	97.2
Marital status^d^	78.8	79.0	80.7	82.6	83.9	84.0	84.5
Education years	15.9(9.7)	16.4(9.5)	17.5(9.2)	18.8(8.8)	18.8(9.4)	19.1(9.0)	20.6(8.9)
Household income ^e^	3481(3044)	4197(3561)	5626(5767)	7473(7692)	8838(11971)	12,421(15667)	15,521(16641)
Community urbanicity^f^	48.3(16.5)	52.3(18.0)	59.3(18.4)	63.1(20.3)	65.0(20.4)	67.5(19.4)	73.2(19.2)
Abdominal obesity	45.1	46.9	49.1	55.1	57.2	59.4	63.4

^a^Values presented as numbers for arbitrary values and as mean ± SD or % for other variables

^b^Gender was a dummy variable coded 100 for males and 0 for females

^c^Ethnicity/race was a dummy variable coded 100 for ethnic Han and 0 for other minorities

^d^Marital status was a dummy variable coded 100 for Married and 0 for others

^e^Per capita net annual household income was calculated at the household level for each survey year and inflated to 2011

^f^Community urbanicity was measured at the community level on a 12-component continuous scale ranging from 0 to 120 with higher values corresponding to higher levels of urbanicity

**Table 2:** Parameter estimates (95% confidence intervals) from mixed effects models predicting of the probability of abdominal obesity over the adults life course

	Model 1	Model 2	Model 3
Age	2.60(2.46,2.74)	1.82(1.49,2.14)	1.508(1.132,1.885)
Age^2^	−0.018(− 0.019,-0.017)	− 0.011(− 0.014,-0.007)	−0.008(− 0.012,-0.004)
Gender^a^	− 0.065(− 0.075,-0.055)	−0.105(− 0.125,-0.086)	−0.100 (− 0.121,-0.080)
1997^b^	4.85(3.66,6.04)	− 6.98(− 16.33,2.37)	−7.67(− 18.09,2.75)
2000	10.98(9.81,12.15)	−17.78(− 27.32,-8.24)	− 21.15(− 32.14,-10.17)
2004	14.48(13.27,15.69)	−15.53(− 25.75,-5.30)	−18.84(− 29.88,-7.80)
2006	15.06(13.83,16.28)	− 11.98(− 22.49,-1.47)	−17.47(− 28.84,-6.10)
2009	19.40(18.17,20.64)	− 9.94(− 20.42,0.54)	−13.17(− 24.50,-1.84)
2011	22.49(21.29,23.70)	−7.10(− 17.16,2.95)	−11.67(− 22.60,-0.74)
1997*Gender		0.027(0.004,0.051)	0.030(0.005,0.056)
2000*Gender		0.039(0.016,0.062)	0.042(0.017,0.068)
2004*Gender		0.036(0.012,0.060)	0.049(0.024,0.074)
2006*Gender		0.041(0.017,0.065)	0.048(0.023,0.074)
2009*Gender		0.044(0.020,0.068)	0.052(0.027,0.077)
2011*Gender		0.071(0.048,0.094)	0.079(0.055,0.103)
1997*Age		0.519(0.100,0.938)	0.497(0.033,0.961)
2000*Age		1.21(0.787,1.631)	1.317(0.838,1.795)
2004*Age		1.257(0.815,1.698)	1.318(0.841,1.795)
2006*Age		1.046(0.598,1.494)	1.200(0.715,1.685)
2009*Age		1.115(0.670,1.560)	1.143(0.660,1.626)
2011*Age		1.126(0.699,1.554)	1.205(0.740,1.670)
1997*Age^2^		−0.006(−0.010,-0.001)	−0.005(− 0.010,-0.0004)
2000*Age^2^		−0.012(− 0.016,-0.008)	−0.013(− 0.018,-0.008)
2004*Age^2^		−0.012(− 0.0170,-0.008)	−0.013(− 0.018,-0.006)
2006*Age^2^		−0.010(− 0.014,-0.005)	−0.011(− 0.016,-0.006)
2009*Age^2^		−0.010(− 0.015,-0.006)	−0.011(− 0.015,-0.006)
2011*Age^2^		−0.011(− 0.015,-0.006)	−0.012(− 0.016, − 0.007)
Ethnicy^c^			0.030(0.003,0.057)
Marital status^d^			0.035(0.022,0.047)
Community urbanicity^e^			0.160(0.130,0.191)
Education years			−0.222(− 0.289,-0.155)
Household income^f^			3.47E^− 4^ (2.23E^− 4^, 4.70E^− 4^)
Community urbanicity*household income			-4.67E^−6^ (− 6.27E^− 6^, − 3.08E^− 6^)
intercept	−40.13(− 43.41,-36.85)	−20.26(− 27.33,-13.19)	−21.94(− 30.45,-13.43)

^a^Gender was a dummy variable coded 100 for males and 0 for females and with 0 as a reference category

^b^Survey year was a dummy variable with 1993 as a reference category

^c^Ethnicity/race was a dummy variable coded 100 for ethnic Han and 0 for other minorities and with 0 as a reference category

^d^Marital status was a dummy variable coded 100 for Married and 0 for others and with 0 as a reference category

^e^Community urbanicity was measured at the community level on a 12-component continuous scale ranging from 0 to 120 with higher values corresponding to higher levels of urbanicity

^f^Per capita net annual household income was calculated at the household level for each survey year and inflated to 2011

Abdominal obesity was less prevalent among males than females [coefficient (95% confidence intervals): − 0.065(− 0.075,-0.055); *P* < 0.001] (Table 2, Model 1), and an interaction between survey year and gender was observed (Table 2, Model 2, Additional file 1: Table S2), which indicated that the period effect was stronger among males than females. Abdominal obesity was more prevalent among ethnic Han Chinese and the married [coefficient (95% confidence intervals): 0.03(0.003, 0.057) and 0.035(0.022, 0.047), respectively; *P* = 0.03 and *P* < 0.001, respectively] (Table 2, Model 3, Additional file 1: Table S3). A higher-level urbanization and higher household income increased the probability of abdominal obesity [coefficient (95% confidence intervals): 0.160(0.130, 0.191) and 3.47E^− 4^ (2.23E^− 4^, 4.70E^− 4^), respectively; all *P* < 0.001], but individuals with a higher-level of education were less likely to develop abdominal obesity [coefficient (95% confidence intervals): − 0.222 (− 0.289, −− 0.155); *P* < 0.001] across adulthood (Table 2, Model 3).

## Discussion

Our longitudinal analysis used data from the 1993–2011 CHNS. This series of surveys, stretching over 18 years, was based on a large nationally representative sample. To our knowledge, this is the first large-scale longitudinal study ever conducted in the whole country to systemically examine socioeconomic disparities in abdominal obesity over the adult life course. The findings showed that prevalence of abdominal obesity increased non-linearly with age over the adult life course. Also, prevalence of abdominal obesity substantially increased with period and cohort, especially for males. Furthermore, women, those of Han ethnicity, the married, and individuals with higher household income or a lower educational level or living in more urbanized communities were more likely to experience abdominal obesity over the adult life course.

No previous studies have examined cohort effects on abdominal obesity in China, and most cross-sectional studies fail to demonstrate a longitudinal trend of abdominal obesity. In longitudinal studies, cohort effects could illuminate the dynamics of abdominal obesity in younger generations [11]. In our longitudinal analyses, abdominal obesity was more prevalent in the younger birth cohorts than older cohorts. In the U.S., the National Health and Nutrition Examination Survey (NHANES) estimated that the Silent Generation (1924–1943) and Generation X (1964–1978) revealed positive cohort effects on abdominal obesity compared with the Baby Boomers (the 1959–1963 birth cohort) [11]. Baby Boomers in the U.S. appeared to experience lower cohort-specific risk of abdominal obesity than older or younger birth cohorts [11]. But abdominal obesity substantially increased with more recent cohorts in China. Compared to the 1931–1940 birth cohort in China, prevalence of abdominal obesity continuously increased and was higher in the 1941–1950, 1951–1960, 1961–1970 and 1971–1980 cohorts.

Consistent with the positive cohort effects, the longitudinal analysis found that prevalence of abdominal obesity also increased consistently during the survey years and age effect was obviously observed in this study. Another study [20] carried out using data from the 1991–2009 China Health and Nutrition Survey found that BMI increased with age and survey years. The two results confirmed that, in terms of age and period effects, the increase in abdominal obesity was matched by an increase in general obesity and WHtR was matched by an increase in BMI, to some extent. Increase in sedentary lifestyle [21, 22], as well as energy intake [23], may play a role in this rise of abdominal obesity. China has experienced rapid changes in physical activity and dietary habits during the past two decades [21–23]. For example, the dietary patterns have changed to high-energy and high-fat foods from predominantly rice and wheat [24]. And younger generations in China tend to have a more sedentary lifestyle [22] and tend to have more meat and dairy product, as well as food heavy with fat and sugar instead of grains, vegetables and fruits [23], which may raise prevalence of abdominal obesity in the younger birth cohorts. Increased prevalence of abdominal obesity among Chinese adults will likely bring higher morbidity and mortality from diabetes [2], metabolic syndrome and cardiovascular diseases [3, 4]. All this implies an emerging and serious public health problem, if there are no effective interventions to prevent and control abdominal obesity.

In our study, prevalence of abdominal obesity was higher among females than males, but the period effect was stronger among men than women. In South Korea [25], U.S. [26], and Sweden [27], males also experienced greater increases in abdominal obesity than females. Some studies showed that increased usage of motor vehicles and associated rise of sedentary lifestyle might have a greater influence on males than females [5, 6], which may contribute to gender differences in abdominal obesity increase. Differences between Han Chinese and ethnic minorities in relation to risk factors, such as differences in dietary patterns [28], may explain lower prevalence of abdominal obesity among ethnic minorities relative to Han Chinese.

Our study showed that urbanization was related to higher prevalence of abdominal obesity, which was consistent with evidence from other developing countries [5, 29]. Urbanization typically decreases levels of physical activity and increases availability of food, particularly fast foods [5]. A study has found that less open space and increased use of motorized transportation in urbanized communities were conducive to physical inactivity [30], which may help explain the increased risk of abdominal obesity among Chinese caught up in the process of urbanization. In this study, individuals with higher educational attainment were less likely to have abdominal obesity across adulthood. Studies in some developed [31] and developing countries [5, 32] have also shown that education was a “protective” factor in relation to obesity. It may be because people with higher education have greater access to and better understanding of information about nutrition, they are more likely to adopt healthier dietary habits and a better lifestyle [6, 8].

What needs to be noted here is that community urbanicity in this study had 12 components, one of which was education. Education as one component of the community urbanicity was related to the individual highest education years to a certain extent. Education as one component of the community urbanicity and the individual highest education years were likely to similarly impact the prevalence of abdominal obesity to a certain extent. But “education” as one component of the community urbanicity was defined as “average education level among adults >21 years old” [19] for the communities, while another “education years” meant the highest education years for individuals. Moreover, the other 11 components (population density, economic activity, housing, diversity, modern markets, traditional markets, communications, transportation infrastructure, social services, sanitation, and health infrastructure) of the community urbanicity [19] may also impact the prevalence of abdominal obesity. For example, transportation infrastructure, one component of community urbanicity, included most common type of road, distance to bus stop, and distance to train stop [19], which might be related to the use of motor vehicles and the rise of physical inactivity, and might further impact the epidemic of obesity in the population [5, 22]. These may be the reason for the results that education was a “protective” factor but community urbanicity was a “risk” factor in relation to abdominal obesity in this study.

In developed countries, age adjusted prevalence of abdominal obesity decreases with income [5, 6]. By contrast, our analysis has found that individuals with higher household income are more likely to experience abdominal obesity. This difference could be due to different stages of social development between developing and developed countries. In high-income countries, the relationship between abdominal obesity and higher income may be attenuated by increased sensitivity about healthy behaviors among better-off individuals and having the financial resources to participate in weight loss efforts [6]. Epidemiological transitions were observed in some middle-income countries where higher epidemic among rich population was revered to in the poor population [5]. In China, household income was an important factor to affect dietary structure and nutrients intake [33]. The traditional Chinese dietary patterns (high carbohydrate, low fat and high dietary fiber) have shifted to greater consumption of meat and animal fat, particularly among wealthier individuals [33]. As the household income increases, Chinese residents’ choices of food will be more abundant, and they may easily access high-fat and energy-dense foods [33].The increased intake of high-energy foods in wealthy residents may lead to weight gains and abdominal obesity. With the rapid increase of China residents’ income, the adverse change of dietary structure may increase the risk of abdominal obesity, general obesity and chronic noncommunicable diseases [33].

The loss of follow-up data is the limitation in this study, and dietary pattern and physical activity are unavailable and not in our statistical analyses. Another limitation is that we were constrained by variables contained in the CHNS. Thus, the key socioeconomic factors we used in our analysis were: gender, ethnicity, marital status, urbanization, educational attainment and per capita net annual household income. As well, we only analyzed the interactions between survey year and gender, between survey year and age, between survey year and age-squared, and between urbanicity and household income. Maybe the collinearity of other variables needs to be taken into consideration in further studies.

## Conclusion

The increasing prevalence of abdominal obesity in China in the past several decades is a function of changing lifestyle of the Chinese people in terms of work, physical activity and diet. Lifestyle changes, in turn, reflect the substantial social and economic transformation of contemporary China. If unchecked, the proportion of the Chinese population becoming overweight or obese will grow and this will become a major public health concern and a challenge to the health care system.

Past studies have amply documented the dire health consequences of obesity. Since the aforementioned social and economic trends in China will likely continue unabated, though perhaps at a less dizzying speed, efforts to halt or even reverse the growing trend of abdominal obesity among the Chinese population are urgently needed. These may include massive undertakings in health promotion and education, particularly concerning diets and physical activity; changes in public policies regarding the food industry, especially the fast food industry; proper labeling of fat, sugar and cholesterol contents in pre-packaged food; and imposing special taxes on “unhealthy” food, such as sugar-loaded soft drinks. Special interventions may also be necessary to target vulnerable populations, such as those we have identified in this study.

## Additional file

Additional file 1: Mixed-effects models (models 1, 2, 3) with age, age-squared, gender, survey year, ethnicity, marital status, education years, community urbanization and per capita net annual household income taken into consideration. (DOCX 32 kb)

## Abbreviations

BMI: Body mass index
CHNS: China Health and Nutrition Survey
WC: Waist circumference
WHtR: Waist-to-height ratio
