# Supplementary material for: Socioeconomic disparities in abdominal obesity over the life course in China
Source: Int J Equity Health. 2018 Jul 5;17:96. doi: 10.1186/s12939-018-0809-x (PMC6034274; doi:10.1186/s12939-018-0809-x)
Supplement: Supplementary file 1 — Mixed-effects models (models 1, 2, 3) with age, age-squared, gender, survey year, ethnicity, marital status, education years, community urbanization and per capita net annual household income taken into consideration. (DOCX 32 kb) [file 12939_2018_809_MOESM1_ESM.docx]

**Additional file 1**

Since a detailed description about the analysis model has been published elsewhere [10, 20], it is briefly introduced in this study. We used the mixed-effects models to estimate the socioeconomic disparities in abdominal obesity across the adult life course. The formulas of mixed effect models (models 1, 2, 3) [10, 20] are as follows:

*Y_it_ = (β_0_ + μ_0i_) + (β_1_ + μ_1i_)Age_it_ + (β_2_ + μ_2i_)Age_it_^2^ + (β_3_ + μ_3i_)Gender_it_ + (β_4_ + μ_4i_)Survey Year_it_ + e_it_*

(1)

*Y_it_ = (β_0_ + μ_0i_) + (β_1_ + μ_1i_)Age_it_ + (β_2_ + μ_2i_)Age_it_^2^ + (β_3_ + μ_3i_)Gender_it_ + (β_4_ + μ_4i_)Survey Year_it_ + (β_5_ + μ_5i_)Gender_ti_ * Survey Year_it_ + (β_6_ + μ_6i_)Survey Year_it_ * Age_it_ + (β_7_ + μ_7i_)Survey Year_it_ * Age_it_^2^ + e_it_*

(2)

*Y_it_ = (β_0_ + μ_0i_) + (β_1_ + μ_1i_)Age_it_ + (β_2_ + μ_2i_)Age_it_^2^ + (β_3_ + μ_3i_)Gender_it_ + (β_4_ + μ_4i_)Survey Year_it_ + (β_5_ + μ_5i_)Gender_ti_ * Survey Year_it_ + (β_6_ + μ_6i_)Survey Year_it_ * Age_it_ + (β_7_ + μ_7i_)Survey Year_it_ * Age_it_^2^ + (β_8_ + μ_8i_)Ethnicy_it_ + (β_9_ + μ_9i_)Marital status_it_ + (β_10_ + μ_10i_)Education years_it_+ (β_11_ + μ_11i_)Urbanicity_it_ + (β_12_ + μ_12i_)Household Income_it_ + (β_13_ + μ_13i_)Urbanicity_it_ * Household Income_it_+ e_it_*

(3)

In the formulas above, key socioeconomic indicators include gender, ethnicity, marital status, education years, community urbanization and household income. Y is the actual measured value of abdominal obesity for subject i at time t; the subjects’ random error is eit; the mean intercept for the inclusion variables of above model are β , β ..., β ; the differences between the intercept of individual i and β , β ..., β are m ,m ..., m . Age effect, period effect, and the impact of socioeconomic disparities in abdominal obesity across the adult lifespan is interpreted by the coefficients in the Equation 1, 2 and 3, respectively.

Table S1. Coefficients (95% confidence intervals) from mixed effects models (model 1) predicting of the probability of abdominal obesity prevalence over the Life Course among China adults.

|  | Coef. | Std.Err. | z | P>\|z\| | [95%Conf. Interval] | |
| --- | --- | --- | --- | --- | --- | --- |
| Age | 2.60054 | 0.07167 | 36.28 | 0.000 | 2.46007 | 2.741011 |
| Age^2^ | -0.01804 | 0.000722 | -25 | 0.000 | -0.019457 | -0.01663 |
| Gender^a^ | -0.06492 | 0.005072 | -12.8 | 0.000 | -0.074857 | -0.05498 |
| 1997^b^ | 4.850961 | 0.607477 | 7.99 | 0.000 | 3.660328 | 6.041593 |
| 2000 | 10.97968 | 0.597959 | 18.36 | 0.000 | 9.8077 | 12.15166 |
| 2004 | 14.47799 | 0.616372 | 23.49 | 0.000 | 13.26992 | 15.68605 |
| 2006 | 15.0574 | 0.624566 | 24.11 | 0.000 | 13.83327 | 16.28152 |
| 2009 | 19.40396 | 0.630168 | 30.79 | 0.000 | 18.16885 | 20.63906 |
| 2011 | 22.4906 | 0.615002 | 36.57 | 0.000 | 21.28521 | 23.69598 |
| intercept | -40.1282 | 1.67464 | -23.96 | 0.000 | -43.41043 | -36.846 |
| ^a^ Gender was a dummy variable coded 100 for males and 0 for femalesand with 0 as a reference category;  ^b^ Survey year was a dummy variable with 1993 as a reference category; | | | | | | |

Table S2.Coefficients (95% confidence intervals) from mixed effects models (model 2) predicting of the probability of abdominal obesity prevalence over the Life Course among China adults.

|  | Coef. | Std.Err. | z | P>\|z\| | [95%Conf. Interval] | |
| --- | --- | --- | --- | --- | --- | --- |
| Age | 1.818084 | 0.165625 | 10.98 | 0.000 | 1.493465 | 2.142703 |
| Age^2^ | -0.01051 | 0.001767 | -5.95 | 0.000 | -0.013972 | -0.00704 |
| Gender^a^ | -0.1054 | 0.009767 | -10.79 | 0.000 | -0.124543 | -0.08626 |
| 1997^b^ | -6.982 | 4.77184 | -1.46 | 0.143 | -16.33464 | 2.370632 |
| 2000 | -17.7834 | 4.868832 | -3.65 | 0.000 | -27.32615 | -8.24068 |
| 2004 | -15.5251 | 5.217597 | -2.98 | 0.003 | -25.75143 | -5.29883 |
| 2006 | -11.9819 | 5.363378 | -2.23 | 0.025 | -22.4939 | -1.46984 |
| 2009 | -9.93918 | 5.34743 | -1.86 | 0.063 | -20.41995 | 0.541595 |
| 2011 | -7.10287 | 5.129566 | -1.38 | 0.166 | -17.15664 | 2.950891 |
| 1997*Gender | 0.027447 | 0.012128 | 2.26 | 0.024 | 0.0036773 | 0.051217 |
| 2000*Gender | 0.039125 | 0.011874 | 3.29 | 0.001 | 0.0158513 | 0.062398 |
| 2004*Gender | 0.036059 | 0.012069 | 2.99 | 0.003 | 0.0124054 | 0.059713 |
| 2006*Gender | 0.041022 | 0.012132 | 3.38 | 0.001 | 0.0172439 | 0.064801 |
| 2009*Gender | 0.043897 | 0.012086 | 3.63 | 0.000 | 0.0202085 | 0.067586 |
| 2011*Gender | 0.070849 | 0.011645 | 6.08 | 0.000 | 0.0480248 | 0.093673 |
| 1997*Age | 0.518566 | 0.213751 | 2.43 | 0.015 | 0.0996209 | 0.93751 |
| 2000*Age | 1.209265 | 0.215208 | 5.62 | 0.000 | 0.7874652 | 1.631066 |
| 2004*Age | 1.256572 | 0.225046 | 5.58 | 0.000 | 0.8154907 | 1.697653 |
| 2006*Age | 1.045831 | 0.228583 | 4.58 | 0.000 | 0.5978161 | 1.493845 |
| 2009*Age | 1.115311 | 0.227113 | 4.91 | 0.000 | 0.6701784 | 1.560443 |
| 2011*Age | 1.126112 | 0.218172 | 5.16 | 0.000 | 0.6985032 | 1.553722 |
| 1997*Age^2^ | -0.00557 | 0.00224 | -2.48 | 0.013 | -0.009957 | -0.00118 |
| 2000*Age^2^ | -0.01206 | 0.002239 | -5.38 | 0.000 | -0.016443 | -0.00767 |
| 2004*Age^2^ | -0.01243 | 0.002303 | -5.4 | 0.000 | -0.016941 | -0.00791 |
| 2006*Age^2^ | -0.00973 | 0.00232 | -4.19 | 0.000 | -0.014276 | -0.00518 |
| 2009*Age^2^ | -0.01023 | 0.002299 | -4.45 | 0.000 | -0.014734 | -0.00572 |
| 2011*Age^2^ | -0.01078 | 0.002212 | -4.87 | 0.000 | -0.015121 | -0.00645 |
| intercept | -20.2613 | 3.607892 | -5.62 | 0.000 | -27.33261 | -13.1899 |
| ^a^ Gender was a dummy variable coded 100 for males and 0 for females and with 0 as a reference category;  ^b^ Survey year was a dummy variable with 1993 as a reference category; | | | | | | |

Table S3.Coefficients (95% confidence intervals) from mixed effects models (model 3) predicting of the probability of abdominal obesity prevalence over the Life Course among China adults.

|  | Coef. | Std.Err. | z | P>\|z\| | [95%Conf. Interval] | |
| --- | --- | --- | --- | --- | --- | --- |
| Age | 1.508208 | 0.192115 | 7.85 | 0.000 | 1.131671 | 1.884746 |
| Age^2^ | -0.008069 | 0.002045 | -3.95 | 0.000 | -0.012078 | -0.004061 |
| Gender^a^ | -0.100479 | 0.010577 | -9.5 | 0.000 | -0.121209 | -0.079749 |
| 1997^b^ | -7.670169 | 5.318292 | -1.44 | 0.149 | -18.09383 | 2.753492 |
| 2000 | -21.15203 | 5.604782 | -3.77 | 0.000 | -32.1372 | -10.16686 |
| 2004 | -18.84004 | 5.633532 | -3.34 | 0.001 | -29.88156 | -7.798516 |
| 2006 | -17.46583 | 5.801419 | -3.01 | 0.003 | -28.83641 | -6.095262 |
| 2009 | -13.17075 | 5.7811 | -2.28 | 0.023 | -24.50149 | -1.839999 |
| 2011 | -11.67014 | 5.57793 | -2.09 | 0.036 | -22.60268 | -0.737597 |
| 1997*Gender | 0.0304538 | 0.013068 | 2.33 | 0.020 | 0.0048408 | 0.0560668 |
| 2000*Gender | 0.0423569 | 0.013126 | 3.23 | 0.001 | 0.01663 | 0.0680838 |
| 2004*Gender | 0.049166 | 0.012819 | 3.84 | 0.000 | 0.0240419 | 0.0742901 |
| 2006*Gender | 0.0480593 | 0.012901 | 3.73 | 0.000 | 0.0227732 | 0.0733454 |
| 2009*Gender | 0.052084 | 0.012857 | 4.05 | 0.000 | 0.0268843 | 0.0772837 |
| 2011*Gender | 0.0788528 | 0.012402 | 6.36 | 0.000 | 0.0545455 | 0.1031601 |
| 1997*Age | 0.4972953 | 0.236745 | 2.1 | 0.036 | 0.0332835 | 0.9613071 |
| 2000*Age | 1.316505 | 0.244255 | 5.39 | 0.000 | 0.8377742 | 1.795236 |
| 2004*Age | 1.317985 | 0.243118 | 5.42 | 0.000 | 0.8414825 | 1.794487 |
| 2006*Age | 1.199672 | 0.24752 | 4.85 | 0.000 | 0.7145415 | 1.684802 |
| 2009*Age | 1.142884 | 0.246277 | 4.64 | 0.000 | 0.6601895 | 1.625579 |
| 2011*Age | 1.205051 | 0.237237 | 5.08 | 0.000 | 0.7400757 | 1.670027 |
| 1997*Age^2^ | -0.005301 | 0.002478 | -2.14 | 0.032 | -0.010158 | -0.000443 |
| 2000*Age^2^ | -0.013354 | 0.002526 | -5.29 | 0.000 | -0.018305 | -0.008404 |
| 2004*Age^2^ | -0.013093 | 0.002494 | -5.25 | 0.000 | -0.017981 | -0.008205 |
| 2006*Age^2^ | -0.011226 | 0.00252 | -4.45 | 0.000 | -0.016166 | -0.006286 |
| 2009*Age^2^ | -0.01054 | 0.002506 | -4.21 | 0.000 | -0.015451 | -0.005628 |
| 2011*Age^2^ | -0.011534 | 0.002415 | -4.78 | 0.000 | -0.016268 | -0.0068 |
| Ethnicy^c^ | 0.1604607 | 0.015754 | 10.19 | 0.000 | 0.1295831 | 0.1913383 |
| Marital status^d^ | 0.0300635 | 0.013862 | 2.17 | 0.030 | 0.0028952 | 0.0572319 |
| Community urbanicity^e^ | 0.0345917 | 0.006465 | 5.35 | 0.000 | 0.0219216 | 0.0472618 |
| Education years | -0.222205 | 0.03428 | -6.48 | 0.000 | -0.289393 | -0.155017 |
| Household income^f^ | 0.0003468 | 6.31E-05 | 5.5 | 0.000 | 0.0002231 | 0.0004704 |
| Community urbanicity*household income | -4.67E-06 | 8.13E-07 | -5.75 | 0.000 | -6.27E-06 | -3.08E-06 |
| intercept | -21.93768 | 4.340728 | -5.05 | 0.000 | -30.44535 | -13.43001 |
| ^a^ Gender was a dummy variable coded 100 for males and 0 for females and with 0 as a reference category;  ^b^ Survey year was a dummy variable with 1993 as a reference category;  ^c^ Ethnicity/race was a dummy variable coded 100 for ethnic Han and 0 for other minorities and with 0 as a reference category ;  ^d^ Marital status was a dummy variable coded 100 for Married and 0 for others and with 0 as a reference category;  ^e^ Community urbanicity was measured at the community level on a 12-component continuous scale ranging from 0–120 with higher values corresponding to higher levels of urbanicity;  ^f^ Per capita net annual household income was calculated at the household level for each survey year and inflated to 2011. | | | | | | |

Table S4. Trajectories of the probability of abdominal obesity prevalence (%) across the life course for 1493 participants with measurements for all 7 surveys among adult, estimated by multilevel mixed effects models stratified by baseline age group (Birth cohort) (Birth cohorts were stratified into 5 groups: Chort 1931-1940, Chort 1941-1950, Chort 1951-1960, Chort 1961-1970, Chort 1971-1980).

|  | Coef. | Std.Err. | z | P>\|z\| | [95%Conf. Interval] | |
| --- | --- | --- | --- | --- | --- | --- |
| Age | 2.503194 | 0.297198 | 8.42 | 0.000 | 1.920697 | 3.085692 |
| Age^2^ | -0.01223 | 0.002852 | -4.29 | 0.000 | -0.01782 | -0.00664 |
| Gender^a^ | -0.47415 | 0.013723 | -34.55 | 0.000 | -0.50105 | -0.44725 |
| Cohort1941-1950^b^ | 8.32789 | 2.202359 | 3.78 | 0.000 | 4.011347 | 12.64443 |
| Cohort1951-1960 | 15.49988 | 2.37179 | 6.54 | 0.000 | 10.85126 | 20.14851 |
| Cohort1961-1970 | 20.7397 | 2.79877 | 7.41 | 0.000 | 15.25421 | 26.22519 |
| Cohort1971-1980 | 34.90811 | 5.525194 | 6.32 | 0.000 | 24.07893 | 45.73729 |
| intercept | -26.4981 | 7.872056 | -3.37 | 0.001 | -41.927 | -11.0692 |
| ^a^ Gender was a dummy variable coded 100 for males and 0 for females and with 0 as a reference category;  ^b^ Birth cohorts were stratified into 5 groups: Cohort 1931-1940, Cohort 1941-1950, Cohort 1951-1960, Cohort 1961-1970, Cohort 1971-1980,with Cohort 1931-1940 as a reference category; | | | | | | |
